# Superficial siderosis

**DOI:** 10.4103/0972-2327.78055

**Published:** 2011

**Authors:** Sameer Vyas, Suresh Giragani, Paramjeet Singh, Anil Bansali, Niranjan Khandelwal

**Affiliations:** Department of Radiodiagnosis, Postgraduate Institute of Medical Education and Research, Chandigarh, India; 1 Department of Endocrinology, Postgraduate Institute of Medical Education and Research, Chandigarh, India

## Introduction

**Figure 1:**
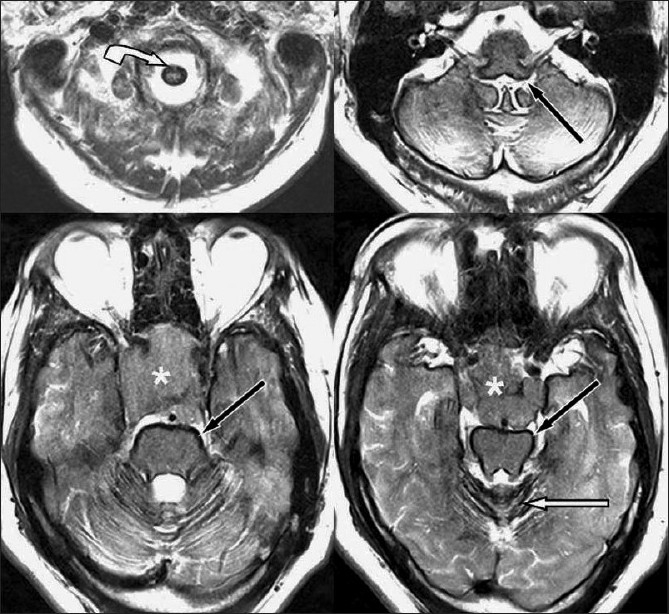
Axial T2WI MR showing intense hemosiderin outlining the dural surfaces, cerebellum (white arrow), brainstem (black arrow), and cervical spinal cord (curved white arrow). In addition, large heterogeneous sellar and suprasellar mass is also seen (asterisk)

**Figure 2:**
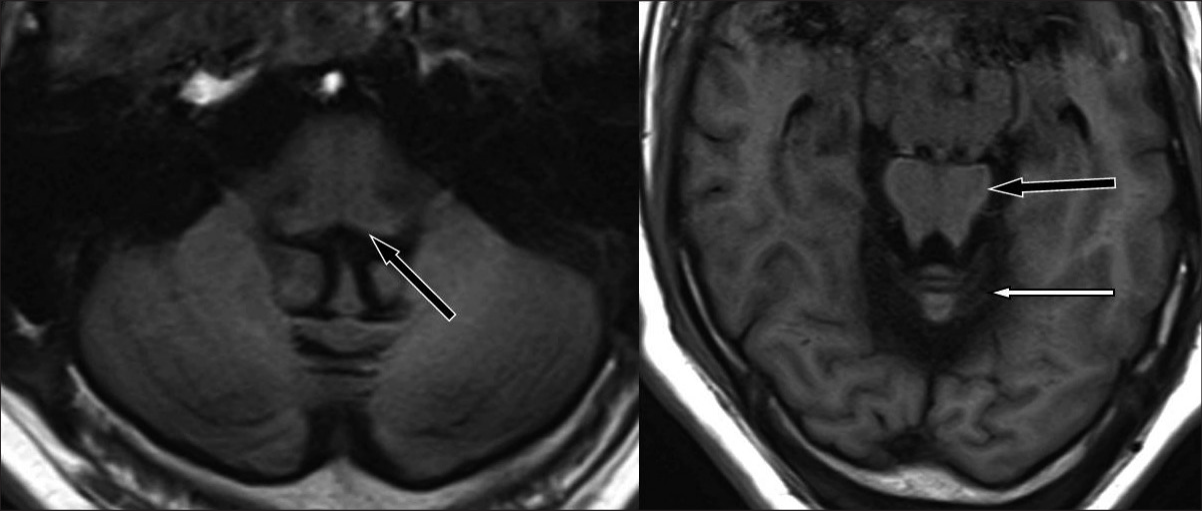
Axial T1WI MR images showing dark outline of the dural surfaces, cerebellum (white arrow) and brainstem (black arrow).

**Figure 3:**
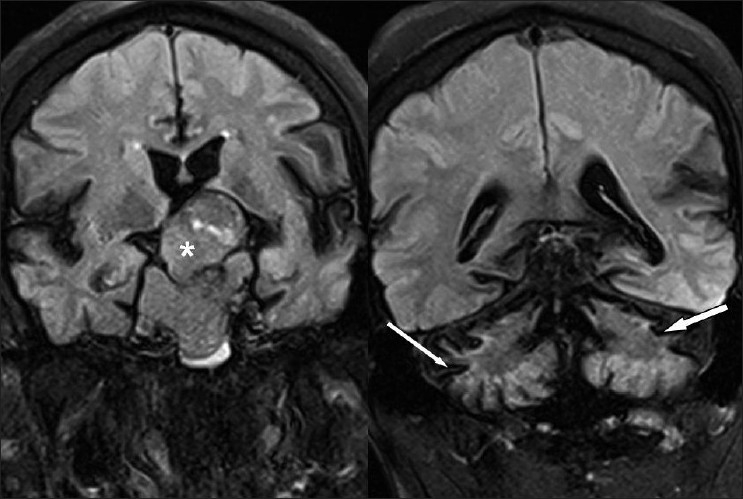
Coronal T2 FLAIR images showing large heterogeneous sellar-suprasellar mass (asterisk) and low signal along and cerebellar surfaces (white arrows)

**Figure 4:**
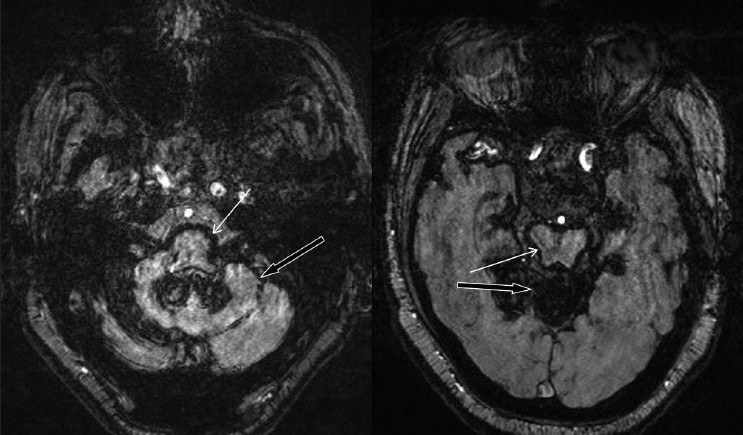
Susceptibility-weighted axial images revealing intense low signal along brainstem (white arrow) and cerebellar surfaces (black arrow) in the posterior fossa

A 40-year-old male presented with complaints of difficulty in walking, decreased cognitive functions, and hardness of hearing since one year. Neurological examination revealed deficiency in cognitive functions of higher intellectual functions. The gait was markedly ataxic with abnormal tests for cerebellar function. Audiometry demonstrated bilateral sensorineural hearing loss. Magnetic Resonance Imaging (MRI) revealed hemorrhagic lesion in sellar-suprasellar region suggestive of pituitary adenoma [Figures 1 and 3]. There was cerebellar atrophy with extensive hypointenities involving leptomeniges predominantly involving structures of posterior fossa on T2 and FLAIR sequences consistent with superficial siderosis [Figures 1–4]. Post-operative histopathology of the sellar-suprasellar lesion showed pituitary adenoma.

Superficial siderosis is a rare chronic progressive neurological dysfunction characterized by classical triad of symptoms consisting of sensorineural hearing loss, cerebellar ataxia, and myelopathy.[1–3] There is deposition of blood breakdown products (hemosiderin) from a source of bleeding in subarachnoid space in the subpial layer of the central nervous system (CNS). Common causes of superficial siderosis include intracranial tumors (21%), head or back trauma (13%), and arteriovenous malformations or aneurysms (9%).[1] Other less common causes include post-surgical changes, brachial plexus injury, amyloid angiopathy, and chronic subdural hematoma. However, despite extensive imaging, a source of bleeding may not be evident in 35% of cases.[1,3] The clinical presentation closely mimics a degenerative cerebellar disorder.[3] Hypointense linear low signal (rim) on T2 images outlining the contours of brain and cranial nerves is the characteristic imaging finding. There is predisposition of CNS structures like cerebellum, brainstem, and spinal cord likely due to the presence of specialized heme absorbing ferritin-producing glial cells in these organs.[2] An intraspinal fluid-filled collection is frequently seen on spine MR imaging in patients with idiopathic siderosis.[3] Treatment of siderosis is identification and treatment of the underlying cause. Surgical removal of source of the bleeding is mainstay in treatment and medical therapy with chelating agents is controversial. With the advent of neuroimaging, this unusual entity can be diagnosed early in the course at which stage it is reversible.
